# Postinjury multiple organ failure in polytrauma: more frequent and potentially less deadly with less crystalloid

**DOI:** 10.1007/s00068-022-02202-8

**Published:** 2023-01-04

**Authors:** Kate L. King, David C. Dewar, Gabrielle D. Briggs, Mark Jones, Zsolt J. Balogh

**Affiliations:** 1 Department of Traumatology, John Hunter Hospital, HRMC, Locked Bag 1, Newcastle, NSW 2310 Australia; 2 University of Newcastle, University Drive, Callaghan, NSW 2308 Australia; 3 Hunter Medical Research Institute, Locked Bag 1000, New Lambton, Newcastle, NSW 2305 Australia; 4 Department of Traumatology, John Hunter Hospital, University of Newcastle, Newcastle, NSW 2300 Australia

**Keywords:** Trauma, Polytrauma, Trauma centre, Multiple organ failure, Traumatic shock, Resuscitation

## Abstract

**Background:**

Recently, retrospective registry-based studies have reported the decreasing incidence and increasing mortality of postinjury multiple organ failure (MOF). We aimed to describe the current epidemiology of MOF following the introduction of haemostatic resuscitation.

**Methods:**

A 10-year prospective cohort study was undertaken at a Level-1 Trauma Centre-based ending in December 2015. Inclusion criteria age ≥ 16 years, Injury Severity Score (ISS) > 15, Abbreviated Injury Scale (AIS) Head < 3 and survived > 48 h. Demographics, physiological and shock resuscitation parameters were collected. The primary outcome was MOF defined by a Denver Score > 3. Secondary outcomes: intensive care unit length of stay (ICU LOS), ventilation days and mortality.

**Results:**

Three hundred and forty-seven patients met inclusion criteria (age 48 ± 20; ISS 30 ± 11, 248 (71%) were males and 23 (6.6%) patients died. The 74 (21%) MOF patients (maximum Denver Score: 5.5 ± 1.8; Duration; 5.6 ± 5.8 days) had higher ISS (32 ± 11 versus 29 ± 11) and were older (54 ± 19 versus 46 ± 20 years) than non-MOF patients. Mean daily Denver scores adjusted for age, sex, MOF and ISS did not change over time. Crystalloid usage decreased over the 10-year period (*p* value < 0.01) and PRBC increased (*p* value < 0.01). Baseline cumulative incidence of MOF at 28 days was 9% and competing risk analyses showed that incidence of MOF increased over time (subdistribution hazard ratio 1.14, 95% CI 1.04 to 1.23, *p* value < 0.01). Mortality risk showed no temporal change. ICU LOS increased over time (subdistribution hazard ratio 0.95, 95% CI 0.92 to 0.98, *p* value < 0.01). Ventilator days increased over time (subdistribution hazard ratio 0.94, 95% CI 0.9 to 0.97, *p* value < 0.01).

**Conclusion:**

The epidemiology of MOF continues to evolve. Our prospective cohort suggests an ageing population with increasing incidence of MOF, particularly in males, with little changes in injury or shock parameters, who are being resuscitated with less crystalloids, stay longer on ICU without improvement in survival.

**Supplementary Information:**

The online version contains supplementary material available at 10.1007/s00068-022-02202-8.

## Introduction

### Background

Postinjury multiple organ failure (MOF) is widely recognised as the most significant contributor to morbidity and late mortality in the severely injured trauma population [1–5]. Reported incidence of MOF is varied, due to multiple definitions, scoring systems and inclusion criteria [6–15]. Similarly, mortality associated with MOF has been reported anywhere from 7.5 to 43% [2, 9, 11]. Our group previously described the epidemiology of MOF in the Australian trauma setting and documented an incidence of MOF of 15% with a 24% mortality rate versus a non MOF mortality rate of 3% [3].

During the study period haemostatic resuscitation became widely accepted, which included streamlining of resuscitation to include reduced crystalloid volumes, the introduction of a massive transfusion protocol encouraging early use of blood products with a 1:1:1 ratio and goal-directed therapy in an attempt to limit prolonged shock and development of MOF [12, 13]. Fundamental changes in resuscitation practices for hemodynamically unstable trauma patients are likely to alter the epidemiology of MOF.

### Objective

Our objective was to describe changes in the epidemiology of MOF over the last 10 years by investigating temporal trends in patient characteristics and outcomes in our institution. We hypothesised that the epidemiology of MOF has changed over time, with a lower incidence and improved outcomes.

## Methods

### Study design and setting

A 10-year prospective population-based cohort study was undertaken at John Hunter Hospital (Newcastle, Australia) from December 2005 until December 2015. In 2005 haemostatic resuscitation guidelines were introduced into our institution and remained unchanged during the study period. The study was approved by The Hunter New England Human Research Ethics Committee, has been performed in accordance with the ethical standards laid down in the 1964 Declaration of Helsinki and its later amendments. John Hunter Hospital is the only Level I Trauma Centre servicing Hunter New England and Mid North Coast Local Health Districts, covering a geographic area of over 143,000 km^2^ with a population over 1 million. It is the busiest Trauma Centre in the state of New South Wales with approximately 4500 trauma admissions annually and 500 severely injured patients, injury severity score (ISS) > 15, admitted per year. During a part of the study period, John Hunter Hospital had the lowest trauma case fatality rate in the state for Level I Trauma Centres, at 9% compared to the state average of 13% [16].

### Participants

All trauma patients admitted into the intensive care unit (ICU) who survived for more than 48 h were considered for the study. The database was designed to identify patients who were most at risk of developing postinjury MOF. The inclusion criteria were patients aged greater than or equal to 16 years with an ISS greater than 15. Exclusion criteria were non-mechanical trauma, head injuries with an abbreviated injury scale (AIS) greater than or equal to 3.

The patients were screened for eligibility upon admission to the ICU by the Trauma Service staff. We recorded Denver scores for the period that patients were in ICU up to 28 days from admission.

### Variables

Prospective data were collected daily in the ICU and stored in our purpose built database, which we developed to collect specific exposure data for our severely injured high-risk population and prospectively maintained since 2005.

Our data included demographics (age, sex, comorbidities) and injury factors (ISS, AIS), shock parameters (blood pressure, highest base excess and lactate at 0–12 and 12–24 h; resuscitation data), haematology parameters (haemoglobin, neutrophil and platelets in the first 24 h after injury and creatinine and bilirubin daily), daily Denver MOF score and outcome data (ICU length of stay (LOS), ventilator days and mortality).

The primary outcome was MOF, which was defined as a Denver MOF Score greater than 3 after the first 48 h and up to 28 days. The score measures physiological parameters after 48 h to exclude reversible responses to trauma and resuscitation (Table 1) [17]. It has been validated and compared to other MOF scoring systems and has been found to be the most specific in describing MOF [10, 14, 15, 18]. The secondary outcomes observed were: ICU LOS, mortality and ventilator days.

**Table 1:** Denver postinjury multiple organ failure score

Dysfunction	Grade 0	Grade 1	Grade 2	Grade 3
Pulmonary; PaO_2_/FiO_2_ ratio	> 250	250–200	200–100	< 100
Renal; Creatinine (µmol/L)	< 159	160–210	211–420	> 420
Hepatic; Total bilirubin (µmol/L)	< 34	34–68	68–137	> 137
Cardiac	No inotropes	Only 1 inotrope at small dose	Any inotrope at moderate dose or > 1 agent at small dose	Any inotrope at large dose or > 2 agents at moderate doses

The MOF daily score is the addition of the worst values for the day for each organ system. MOF is defined as a score > 3 [19]

The variables of primary clinical interest were Denver Scores, MOF, mortality, ICU length of stay and ventilator days over the 10-year study period. Given the potential for confounding and effect modification, we initially explained association between patient variables.

### Data sources and measurement

Demographic continuous variables were summarised using means and standard deviations (for symmetrical distributions) and medians and inter-quartile range (for skewed distributions). We summarise categorical variables with frequency of patients and percentage of total. Characteristics of the MOF patients were compared to non MOF patients using t-test/Kruskal–Wallis rank sum test for continuous variables and Chi-squared test for categorical variables.

We examined temporal trends over the study period in the distributions for age, gender, injury severity and the quantity of crystalloids and PRBC used. We have provided a more detailed description of our statistical methods as supplementary material within Supplementary Information section.

We consider a *p* value of < 0.05 to be statistically significant. All statistical analysis was programmed using Stata version 14 and R version 3.5.1.

## Results

For clarity of expansion, we provide a narrative description of the results for the clinical outcomes, but only a brief summary of our exploratory analyses in this section.

### Dataset

**Fig. 1:**
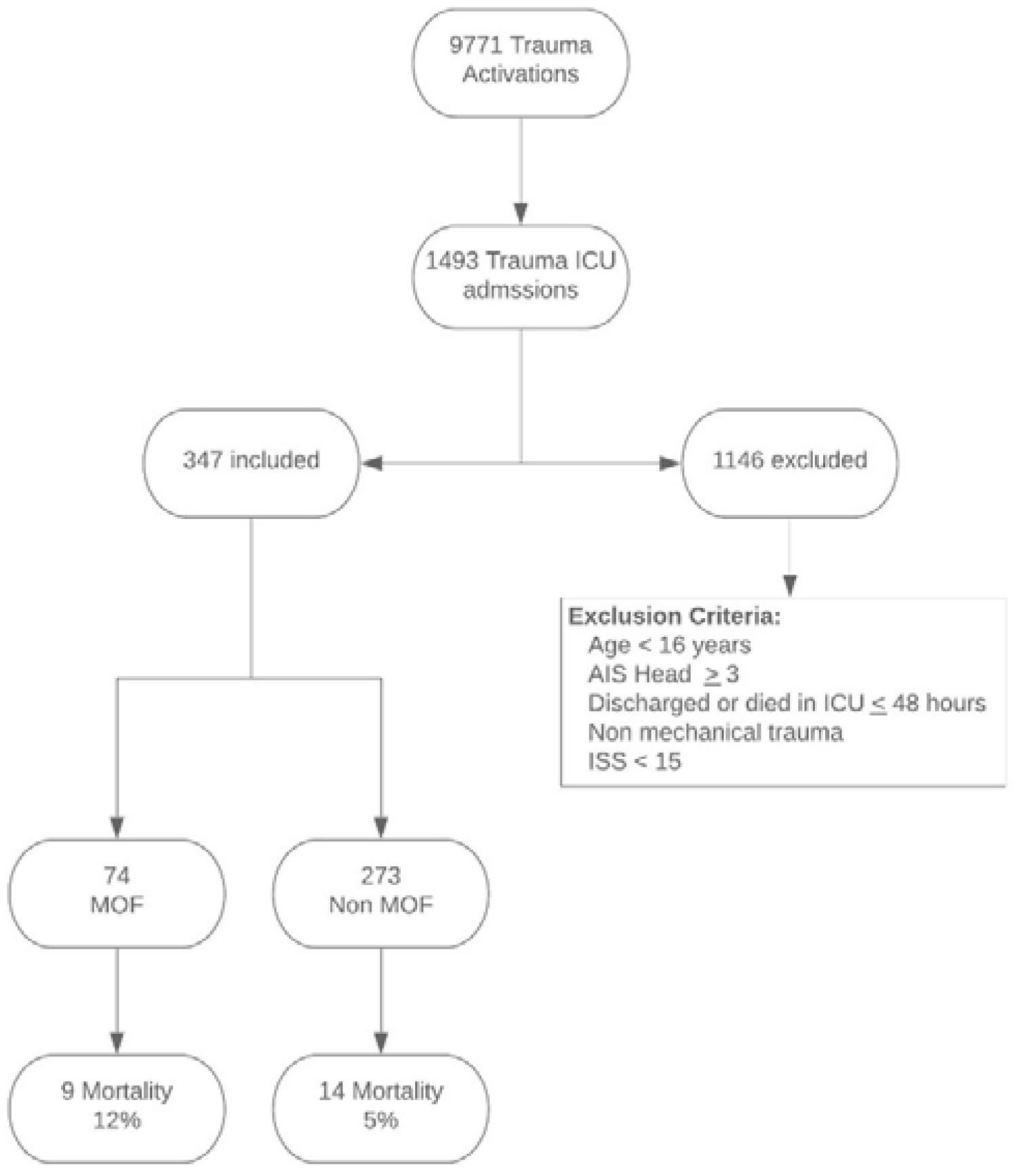
Study population

In total there were 9771 trauma team activations during the study period, 1493 patients were admitted to the ICU, 1146 patients were excluded for head injury with an AIS score ≥ 3, paediatric, non-mechanical trauma discharge from ICU or death before 48 h. This left 347 patients eligible for entry into the institutional MOF database of which 74 patients (21% incidence) developed post-injury MOF, 9 (12%) of the MOF patients died and 14 (5.1%) of the non-MOF patients died (Fig. 1).

The sample population mean (SD) age over the study period was 48 (20.2) years, increasing by 0.78 (95% CI 0.02 to 1.54, *p* value < 0.05) years per annum. MOF patients were older than non-MOF patients. Males had higher representation (overall mean 71%) in the sample, increasing over the study period by around 2% per year with univariate regression (risk ratio (RR) 1.02 95% CI 1.00 to 1.05, *p* value = 0.5). Males were also more frequent among MOF patients (81%) than non-MOF patients (68%). The median injury severity score was 29 and this did not change over the study period (RR. 0.99, 95% CI 0.98 to 1.01, *p* value = 0.3).

The volume of crystalloid given in the first 12 h after injury (accounting for ISS, age sex and PRBC) reduced by around 5% per annum (RR 0.95, 95% CI 0.93 to 0.96, *p* value < 0.01) over the study period. Similarly, the 12–24 h crystalloid use (accounting for ISS, age sex and PRBC) showed a 6.3% reduction per annum over the study period (RR 0.93, 95% CI 0.92 to 0.95, *p* value < 0.01).

The use of PRBC in the first 12 h of care (accounting for ISS, age, sex and crystalloid use) increased over the study period by around 7% per annum. However, use in the 12 to 24 h period (accounting for ISS, age, sex and crystalloid use) showed no change (RR 0.99, 95% CI 0.92 to 0.95, *p* value < 0.01).

Table 2 provides further descriptive statistics on sample characteristics stratified by MOF versus non-MOF.

**Table 2:** Patient demographic summary statistics for selected characteristic

	Total	MOF	Non-MOF	*p* value
*N*	347	74	273	
Age (mean (sd))	47.9 (20.2)	54.6 (19.5)	46.0 (20.0)	< 0.01
Sex = female/male (%)	99/248 (29/71)	12/62 (16/84)	87/186 (32/68)	0.01
Death = yes/no (%)	23/324 (7/93)	9/65 (12/88)	14/259 (5/95)	0.06
Injury Severity Score (mean (sd))	29.7 (11.2)	31.9 (11.2)	29.1 (11.2)	0.06
New Injury Severity Score (mean (sd))	35.4 (11.8)	38.9 (11.9)	34.5 (11.6)	< 0.01
Blunt mechanism = yes/no (%)	328 (95)	71 (96)	257 (94)	0.5
Cardiac Denver Score (mean (sd))	0.2 (0.3)	0.5 (0.3)	0.1 (0.3)	< 0.01
Hepatic Denver Score (mean (sd))	0.1 (0.4)	0.4 (0.5)	0.1 (0.2)	< 0.01
Renal Denver Score (mean (sd))	0.2 (0.5)	0.6 (0.9)	0.0 (0.1)	< 0.01
Respiratory Denver Score (mean (sd))	0.7 (0.7)	1.2 (0.6)	0.6 (0.6)	< 0.01
Total Denver Score (mean (sd))	1.2 (1.1)	2.7 (1.2)	0.8 (0.7)	< 0.01
Maximum total Denver Score (mean (sd))	2.6 (2.0)	5.5 (1.8)	1.8 (1.3)	< 0.01
Denver Days (mean (sd))	9.6 (7.0)	17.3 (7.3)	7.5 (5.2)	< 0.01
Los ICU (median [iqr])	8.0 [4.0, 12.0]	16.0 [11.0, 25.0]	6.0 [4.0, 9.0]	< 0.01
Ventilator Days (median [iqr])	5.0 [3.0, 10.0]	13.5 [9.2, 20.0]	4.0 [2.0, 7.0]	< 0.01
Apache 2 Score (mean (sd))	16.9 (7.3)	21.2 (7.1)	15.8 (6.9)	< 0.01
0–12 h Base Deficit (mean (sd))	− 4.8 (5.1)	− 5.3 (6.2)	− 4.7 (4.8)	0.4
12–24 h Base Deficit (mean (sd))	− 2.2 (3.4)	− 3.0 (3.6)	− 2.0 (3.3)	0.03
0–12 h Lactate Level (mean (sd))	3.8 (3.1)	4.7 (3.4)	3.6 (2.9)	0.01
12–24 h Lactate Level (mean (sd))	1.9 (1.5)	2.3 (1.8)	1.8 (1.4)	0.01
0–12 h Units of PRBC (mean (sd))	3.6 (5.0)	4.8 (5.3)	3.3 (4.9)	0.02
12–24 h Units of PRBC (mean (sd))	0.9 (2.8)	1.7 (4.6)	0.7 (2.0)	0.01
0–12 h Units of Crystalloid (mean (sd))	4.3 (2.8)	4.7 (2.8)	4.1 (2.8)	0.12
12–24 h Units of Crystalloid (mean (sd))	2.4 (1.4)	2.3 (1.3)	2.4 (1.5)	0.73

### Clinical outcomes

#### Denver score

The mean daily Denver scores increased with increasing decadal age (0.08, 95% CI 0.04 to 0.12, *p* value < 0.01), with increasing injury severity (0.01, 95% CI 0.00 to 0.20, *p* value < 0.05) and with increasing 0–12 h PRBC (0.03, 95% CI 0.01 to 0.05, *p* value < 0.01). From the four components of the Denver MOF score only the cardiac score (0.02, 95% CI 0.01 to 0.03, *p* value < 0.01) showed increase during the study period.

### Multiple organ failure

**Fig. 2:**
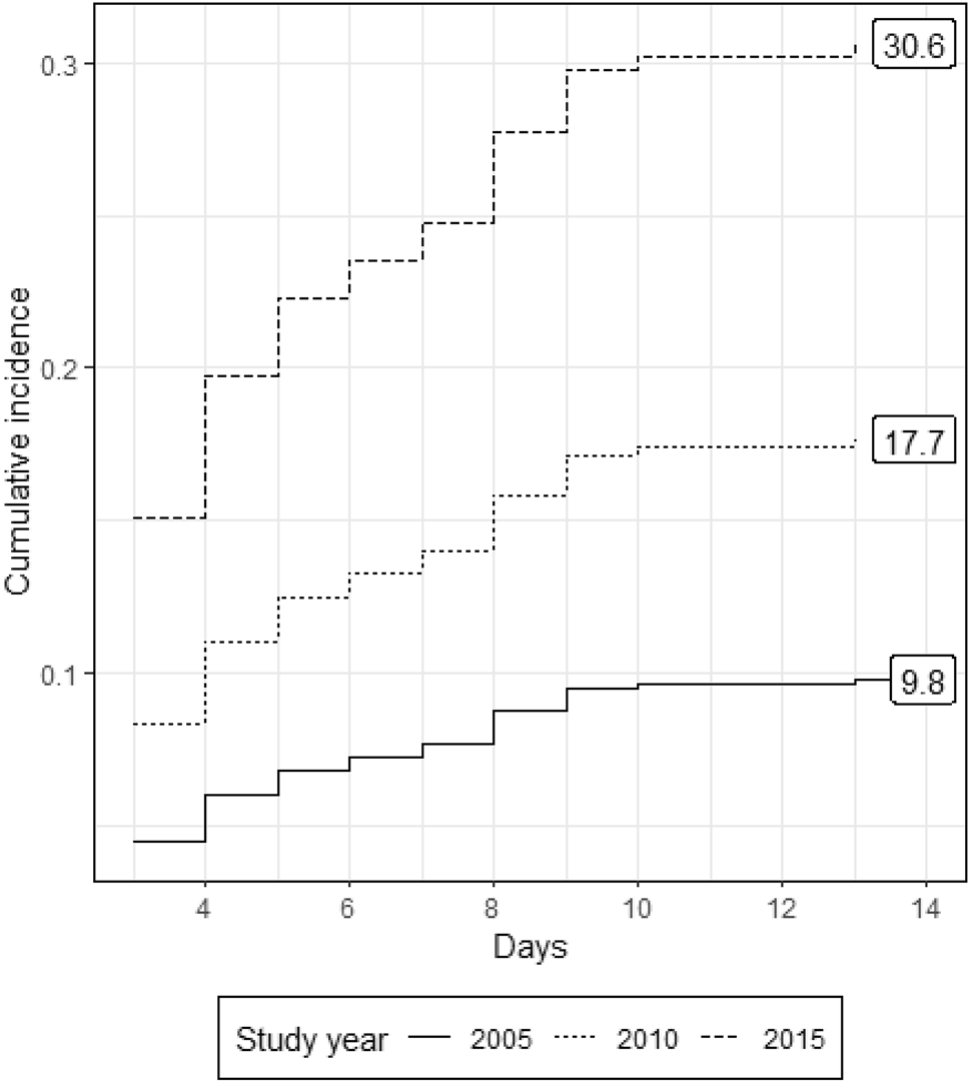
Cumulative incidence of MOF by admission year (2005, 2010 and 2015)

The observed proportion of MOF patients increased over the study period from 14% in the first year to 33% in 2015. This observed increase was supported by a binomial regression (log link) that regressed the occurrence of MOF on the covariates of age, sex and injury severity. The model suggested an increasing annual temporal trend (RR 1.13, 95% CI 1.05 to 1.22, *p* value < 0.01) (Fig. 2).

### In-hospital mortality

Of the 347 patients, 23 (7%) died. A binomial model regressing death on admission year with covariates for age, sex and injury severity was inconclusive for temporal trends (RR 1.07, 95% CI 0.93 to 1.23, *p* value = 0.4). However, the model did show a significant association between increased age and mortality (RR 1.06, 95% CI 1.03 to 1.08, *p* value < 0.001) and higher severity of injury was not associated significantly with mortality (RR 1.03, 95% CI 0.99 to 1.05, *p* value = 0.16).

**Fig. 3:**
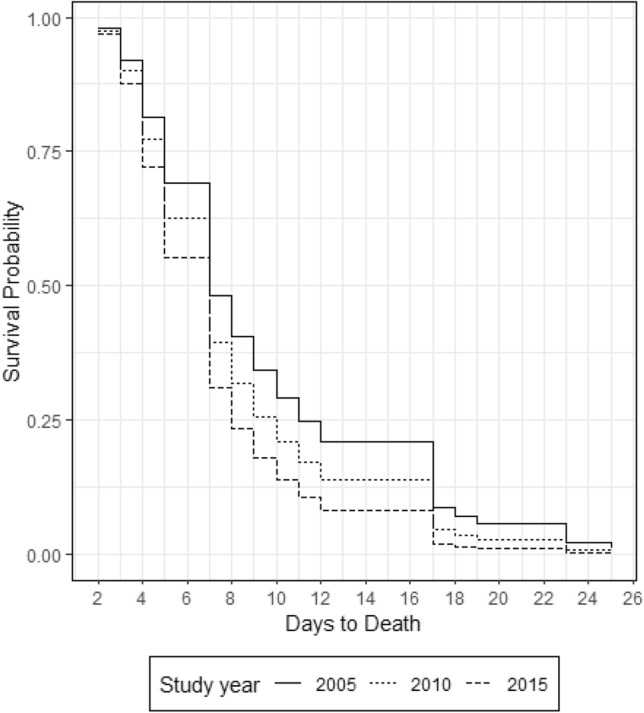
Survival probability (time to death) by admission year (2005, 2010 and 2015)

Similarly, a Cox proportional hazard regression for days to mortality with covariates for year of admission, MOF, decadal age, sex and injury severity suggested no association between time to death and the study year (hazard ratio (HR) 1.00, 95% CI 0.80 to 1.31, *p* value = 0.9). However, the hazard ratio for the MOF term was 0.3 (95% CI 0.1 to 0.8, *p* value = 0.024) indicating that those with MOF had an extended time to death (Fig. 3).

### ICU LOS

Of the 347 patients in ICU, 326 were discharged (median LOS 7 days) and 21 died (median LOS 9 days).

### Ventilator-free days

**Fig. 4:**
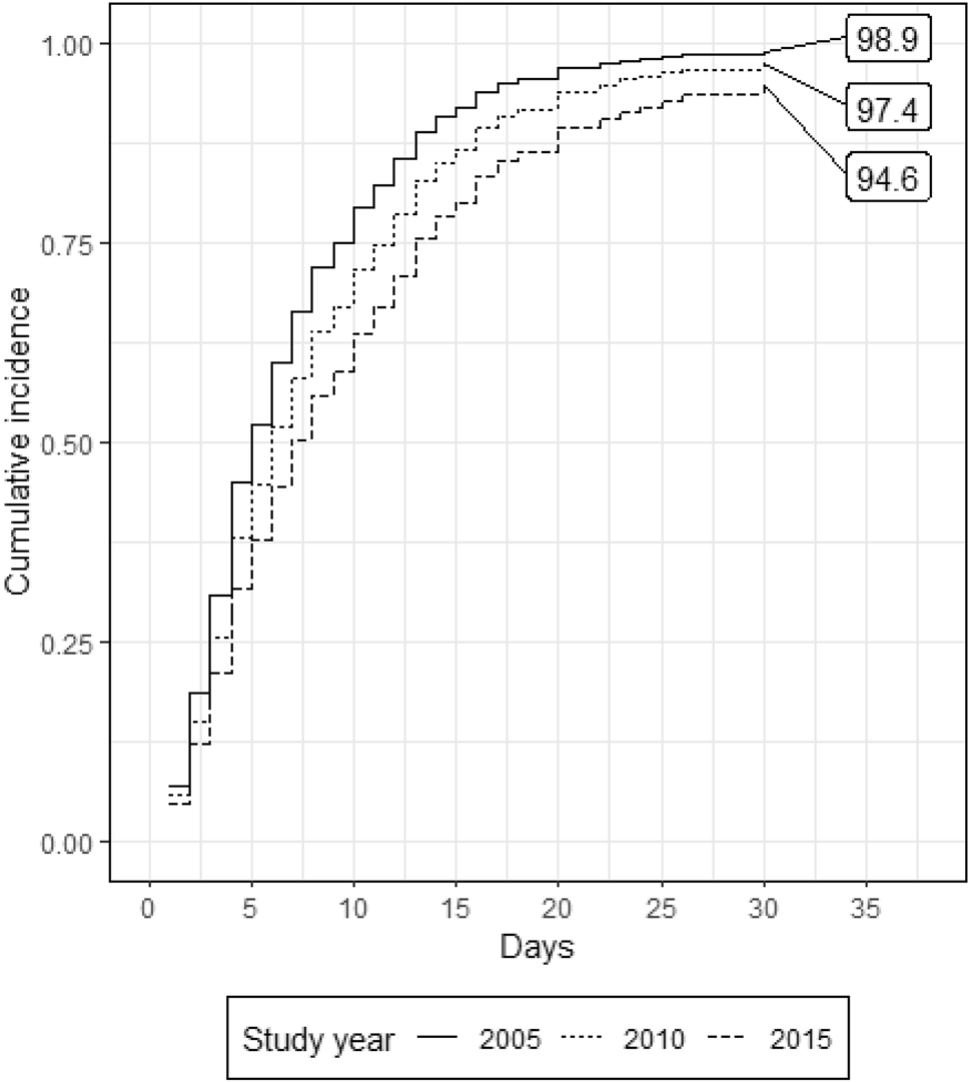
Cumulative incidence of extubation days in the presence of competing risk of death for admission years—2005, 2010 and 2015

Our competing risk analysis for extubation (with competing risk of death) regressed the subdistribution hazard of extubation on year of admission, MOF, age, sex and injury severity. The subdistribution hazard ratio year of admission was 0.96 (95% CI 0.92 to 1.00, *p* value < 0.05), suggesting a decrease in the cumulative incidence (probability) of extubation for each year increase over the study period. In practical terms this meant we saw an increase in ventilator days over time. We estimated the cumulative incidence was 98% in 2005 decreasing to 89% by 2015 (Fig. 4).

## Discussion

### Key findings

Our prospective cohort study identified 21% incidence of MOF over 10 years among high-risk major trauma patients admitted to the ICU, the incidence showed significant increase during the study period. We observed greater risk of MOF with older, male patients and a higher injury severity. Furthermore, both PRBC and crystalloid usage were positively correlated with incidence of MOF. Whilst mortality did not change over the decade, MOF patients required the utilisation of more hospital resources reflected in longer ICU and ventilator days.

Any discussion of changes in the epidemiology of MOF requires an understanding of the multiple definitions used across the world. Currently there is no consensus on defining postinjury MOF and there is no consensus on which patients are included into the high-risk study cohort [14, 15]. Our data are most comparable to the Denver group’s data, based on inclusion criteria and definition of MOF through the use of the same scoring system yet we are demonstrating very different changes in the epidemiology of MOF.

Our reported increasing incidence of MOF does not align with recent reports out of North America [1, 19]. The low incidence described has been supported by benchmarking data across both trauma and non-trauma centres in the USA [20]. Other studies have described an increasing incidence of MOF [2, 21]. Froehlich et al. report an increasing incidence 24.6% in 2002 to 31.5% in 2011 but they utilised different scoring system and inclusion criteria, and furthermore, they also described a decreasing mortality rate [2]. Given the nature of these studies and the relatively high potential for confounding from unobserved variables, variation of this kind is not overly surprising.

Observed mortality in our MOF population has decreased from our historical 24 to 12% in our current cohort [3]. This is in contrast to the temporal mortality trends described by Sauaia et al. where their MOF-related mortality rate has increased over time [1]. We note that changes in our resuscitation regime over the same time period that were associated with the risk of MOF but we did not detect an association with mortality. Additionally, the changing demographics of our patient population that were not measured in this study may account for the impact on incidence. As with our study, previous work has demonstrated increasing age and male gender are associated with an increasing incidence of MOF [15, 22–25].

The Australian population is ageing with the median Australian age now 37 years compared to 34 years in 2005, 15% of the Australian population are older than 65 years [26]. Our high-risk study population reflected this, with the population ageing 0.78 years per calendar year which is in keeping with previous studies. Increasing age may also be contributing to the increased ventilator days and ICU LOS as suggested by our analyses. Although there was no difference in the MOF subgroup for ageing, the MOF population remains older than the non-MOF group. Age is also a risk factor for both increased ventilator days and ICU LOS [27].

The contributions of organs that fail in MOF have altered over the 10-year study period. In 2015 MOF patients are likely to have a higher component of cardiac failure compared to 2005. The more frequent cardiac failure could be due to the older population and to potentially more liberal use of inotropes instead of crystalloid boluses. The component of the score made up by hepatic, respiratory and renal failure did not change significantly over the study period. The increased Denver cardiac scores may be contributing to the upward trend in both ventilator days and ICU LOS.

Haemostatic resuscitation was introduced into our institution at the beginning of 2005, this included the introduction of a massive transfusion protocol and goal-directed therapy [28]. The concept of haemostatic resuscitation, recognising the therapeutic end points as important, no longer targeting blood pressure but making an accurate assessment and then supporting organ perfusion whilst assessing and correcting coagulopathy and gaining anatomical control of any bleeding [28–31]. There was a gradual alignment with the pre-hospital services providers as they moved towards limiting crystalloid resuscitation.

Our use of PRBC increased in the first twelve hours after injury by 7% per calendar year over the study period, due to the early empiric blood and component-based therapy. Not surprisingly PRBC increased with increasing injury severity. These are similar volumes described in the international papers [1, 2].

Studies that have used data from the Glue Grant Database demonstrated that aggressive early crystalloid dose-dependent resuscitation impacted on the incidence of acute lung injury (ALI), acute respiratory distress syndrome (ARDS), MOF, abdominal and extremity compartment syndrome [22, 23]. Minei et al. describe large volumes, 10.5 L of crystalloid was the mean volume given in the 0–12 h time frame [22]. Our volumes have never reached those limits with a mean volume of 4.2 L in the whole cohort and 4.7 L in the MOF group in the first 12 h postinjury. The use of crystalloids is certainly a modifiable factor [31], and the differences in volumes used in these trauma centres may go some way in explaining the differences in the epidemiology we have described.

### Limitations

This study was conducted in a single centre Trauma Centre and differences in haemostatic resuscitation and ICU admission policies, and ICU case mix may have influenced our findings and limit direct comparison. Excluding severe head injuries may change our true incidence. Observation of the proportion of in-hospital mortality over the study period was compromised due to the low mortality rate. There are strengths and weaknesses in all of the validated MOF scoring systems. Using the Denver MOF Score whilst specific has less sensitivity than some of the other scoring systems such as SOFA.

## Conclusion

Our ten-year prospective population-based study has demonstrated an increase in the incidence of MOF in a significantly older cohort, which was resuscitated with less crystalloids. In our gradually older population, the mortality of MOF did not change over time but it requires increasingly more hospital resources.

### Supplementary Information

Below is the link to the electronic supplementary material.

Supplementary file1 (DOCX 23 KB)

## Data Availability

No data was made available on public domain related to this study, on specific request data sharing is possible after ethics committee approval.
