# Supplementary material for: Postinjury multiple organ failure in polytrauma: more frequent and potentially less deadly with less crystalloid
Source: Eur J Trauma Emerg Surg. 2023 Jan 4;50(1):131–8. doi: 10.1007/s00068-022-02202-8 (PMC10923957; doi:10.1007/s00068-022-02202-8)
Supplement: Supplementary file 1 — Supplementary file1 (DOCX 23 KB) [file 68_2022_2202_MOESM1_ESM.docx]

**Supplementary Information**

**POSTINJURY MULTIPLE ORGAN FAILURE IN POLYTRAUMA: MORE FREQUENT AND POTENTIALLY LESS DEADLY WITH LESS CRYSTALLOID.**

**Journal: European Journal of Trauma and Emergency Surgery**

Kate L King RN MN^a,b^, David C Dewar FRACS, MBBS B(med)Sc, Ph.D.^a,b^ Gabrielle D Briggs Ph.D., BBiomed(Hons)^a^,Mark Jones MBiostat^c^ Zsolt J Balogh MD Ph.D^a,b^.

a.John Hunter Hospital. b.University of Newcastle. c.Hunter Medical Research Institute

Full postal address

1. Department of Traumatology

John Hunter Hospital

Locked Bag 1, HRMC

NSW 2310 Australia

b. University of Newcastle

University Drive
Callaghan NSW 2308 Australia.

1. Hunter Medical Research Institute, Newcastle, NSW, Australia

Locked Bag 1000
New Lambton NSW 2305

**Corresponding Author:** Zsolt J Balogh, MD, PhD, FRACS

Department of Traumatology, John Hunter Hospital, University of Newcastle

Newcastle NSW 2300, AUSTRALIA

Tel +612 4921 4259

Fax: +612 4985 5545

Email: [Zsolt.Balogh@health.nsw.gov.au](about:blank)

**Extended methods and results on exploratory Analyses on Potential Confounders / Effect Modifiers**

Extended Methods

We used linear regression to model temporal trends in patient age and mean daily Denver scores. We note that while the individual scores are ordinal, regression of the mean daily Denver scores on covariates is justified under the central limit theorem. We used generalised linear models (GLM) for modelling proportions other variables of interest. Specifically, we used binomial regression (log link to give results in terms of risk ratios (RR)) to assess whether the proportion of males to females had changed over time by representing sex as a binary variable and introducing covariates for year of study etcetera. For the quantity of units of crystalloids used we adopted a Gamma GLM with log link we used Poisson regression (log link) to model temporal trends in PRBC and ISS. Calibration was examined via the Hosmer and Lemeshow goodness of fit and discrimination using a C-statistic (area under the curve).

For the clinical events of interest that were subject to competing risks (MOF, ICU LOS, ventilator days) we apply both generalised linear models and Fine and Grey competing risk regression [1]. The Fine Grey model explicitly addresses the complication of competing risks, whereby the ability of one event to occur e.g. MOF, is contingent on another not having occurred e.g. death. We produced calmative incidence curves and report the subdistribution hazard ratios (SHR), 95% confidence intervals, p-values, cumulative incidence, report model AIC as a relative measure if goodness-of-fit when applicable. We checked the proportional hazard and linearity assumptions in the Fine and Grey models using cumulative residuals as proposed by Li et al ^2^. We modelled in-hospital mortality using the Cox proportional hazard model.

The following results relate to characteristics that represent potential confounders and effect modifiers.

**Age**

The mean (SD) patient age over the study period was 48 (20.2) years. On average, MOF patients were older than the non-MOF patients (**Table 1**). Univariate linear regression suggested age had increased by 0.78 (95% CI 0.02 to 1.54, p-value < 0.05) years per calendar year over the 10 year period.

**Sex**

Males made up 71% of the overall study population. The MOF cohort had a statistically significant greater proportion of males compared to the non-MOF patients (84% vs. 68% respectively, Chi-square = 6.25, p-value = 0.01) (**Table 1**). Univariate binomial regression (log link) suggested that the proportion of male admissions had increase by 2% (RR 1.02, 95% CI 1.00 to 1.05, p-value = 0.05) per calendar year. Introducing a covariate for MOF showed a statistically significant association between the proportion of males and MOF status (RR 1.21, 95% CI 1.05 to 1.37, p-value < 0.01) but the model was then inconclusive regarding the temporal trend (RR 1.02, 95% CI 1.00 to 1.04, p-value = 0.1). An interaction term to model differential temporal trends by MOF was non-significant (est. 1.01, 95% CI 0.96 to 1.06, p-value = 0.7).

**Crystalloids**

Crystalloid use in the first 12 hours of care adjusted for injury severity, age, sex and PRBC showed a reduction in usage by approximately 5.4% (est. 0.95, 95% CI 0.93 to 0.96, p-value < 0.01) per calendar year over the 10-year period. As expected, crystalloid usage was elevated with increasing ISS (est. 1.01, 95% CI 1.00 to 1.01, p-value = 0.08) and increasing units of PRBC in the 0-12 hour period (est. 1.05, 95% CI 1.04 to 1.06, p-value < 0.01), but decadal age and sex estimates were inconclusive. A sex (female referent) by age interaction term was supported by the data (est. 1.08, 95% CI 1.02 to 1.15, p-value < 0.01) with both the decadal age and sex terms significant at the 0.05 level. However, the resulting model did not improve fit (AIC reduced from 1632 to 1631) and no further two-way interactions were supported by the data.

Crystalloid use in the 12-24 hours of care adjusted for ISS, age, sex and PRBC, showed a reduction in usage by approximately 6.3% (est. 0.93, 95% CI 0.92 to 0.95, p-value < 0.01) per calendar year over the 10-year period. Crystalloid usage was elevated with increasing units of PRBC in the 12-24 hour period (est. 1.04, 95% CI 1.02 to 1.07, p-value < 0.01), but was inconclusive for changes in decadal age, sex and injury severity. An interaction term between sex (female referent) and PRBC volume was supported by the data (est. 0.95, 95% CI 0.90 to 1.00, p-value = 0.02) but resulted in negligible change to the AIC (1165 to 1164).

**PRBC**

PRBC use in the first 12 hours of care adjusted for ISS, age, sex and crystalloid use showed an increase of approximately 7% (est. 1.07, 95% CI 1.02 to 1.13, p-value < 0.01) per calendar year over the 10-year period. PRBC usage increased with injury severity (est. 1.02, 95% CI 1.01 to 1.03, p-value < 0.01), and increasing units of Crystalloids in the 0-12 hour period (est. 1.16, 95% CI 1.13 to 1.19, p-value < 0.01), but the association between PRBC usage and age and sex were all inconclusive. An interaction term between age and sex (female referent) was supported by the data (est. 0.87, 95% CI 0.76 to 0.98, p-value < 0.01) and had a material impact on the AIC (2199 reduced to 2178).

PRBC use in the 12 to 24 hour period of care adjusted for ISS, age, sex and units of crystalloids in the 12 to 24 hour period was inconclusive with regards to an annual temporal trend (est. 0.99, 95% CI 0.90 to 1.09, p-value = 0.9). However, elevated PRBC use was associated with increasing ISS (est. 1.03, 95% CI 1.01 to 1.05, p-value < 0.01) and crystalloid usage in the 12 to 24 hour period (est. 1.26, 95% CI 1.09 to 1.44, p-value < 0.01) and inconclusive with regards to sex. Age did not show association with PRBC usage and no two-way interactions were supported by the data.

**Injury Severity Score**

Poisson regression for ISS with covariates for year, age and sex was inconclusive with regards to temporal trends (est. 0.99, 95% CI 0.98 to 1.01, p-value = 0.3). The median ISS was 29.

MOF

A related analysis of MOF considered the period of time to MOF (with death as a competing risk) modelled in terms of the subdistribution hazard that is assumed by a Fine and Gray model. This model included covariates for year of study, age, sex and injury severity and suggested that a unit increase in admission year was associated with a 1.14 (SHR 1.14, 95% CI 1.04 to 1.23, p-value < 0.01) factor increase in the subdistribution hazard of MOF. The result implies that the instantaneous hazard of MOF increased over the study period as did the incidence (probability) of MOF.

1. Fine J. P. and Gray R. J. A proportional hazards model for the subdistribution of a competing risk. *J. Am. Stat. Assoc.* 199 Jun; 94(446): 496-509
2. Li J, Scheike TH, Zhang MJ. Checking Fine and Gray subdistribution hazards model with cumulative sums of residuals. *Lifetime Data Anal*. 2015 Apr; 21(2):197-217
